# Negative pressure wound therapy for combat-related extremity vascular injuries: clinical experience from the war in Ukraine

**DOI:** 10.1186/s13049-025-01502-3

**Published:** 2025-11-25

**Authors:** Iurii I. Sivash, Boris M. Koval

**Affiliations:** 1 National Military Medical Clinical Center “Main Military Clinical Hospital”, Kyiv, Ukraine; 2 Bogomolets National Medical University, Kyiv, Ukraine

**Keywords:** NPWT, Combat extremity vascular trauma, Limb salvage, Soft-tissue defect, War in Ukraine

## Abstract

**Background:**

Extremity vascular injuries are among the most challenging problems in military surgery. They are frequently accompanied by extensive soft tissue loss and heavy contamination, which increases the risk of infection and limb loss. Although negative pressure wound therapy (NPWT) is widely used in civilian practice, its role in combat vascular injuries remains unclear. The war in Ukraine provided an opportunity to evaluate NPWT as part of staged surgical care under modern battlefield conditions.

**Methods:**

We retrospectively reviewed 85 service members with severe combat-related extremity vascular injuries admitted to a Role IV facility in 2022. Among these patients, 69/85 (81.2%) had extensive soft-tissue defects overlying vascular reconstructions and received NPWT; this subgroup constituted the analytic cohort. A standardised two-layer NPWT technique was used: an inner nonadherent barrier/PVA sponge directly over the reconstruction site and an outer polyurethane foam connected to continuous –70 to –80 mmHg. The dressing was changed every 3–4 days. The outcomes included infectious complications, erosion-related bleeding, arterial thrombosis, secondary amputation, the method of definitive wound closure, and the length of stay.

**Results:**

The mechanisms of injury were mine blast (71%), gunshot (23%), and other explosive trauma (6%). Combined arterial–venous injuries occurred in 40% (*n* = 28/69), fractures in 42% (*n* = 29/69), and primary wound contamination in 57% (*n* = 39/69) of the patients. Definitive closure was achieved by primary approximation in 75.4% (*n* = 52/69), skin grafting in 17.4% (*n* = 12/69), and flap techniques in 4.3% (*n* = 3/69). Complications occurred in 27.5% (*n* = 19/69): erosion-related bleeding (13%, *n* = 9/69), arterial thrombosis (8.7%, *n* = 6/69), and infection (5.8%, *n* = 4/69). Erosion-related bleeding clustered in two risk windows: days 7–10 and 18–30. Secondary amputation was required in 2.9% (*n* = 2/69); in-hospital mortality was 0%.

**Conclusions:**

A two-layer NPWT protocol at –70 to –80 mmHg was a safe and effective adjunct in the staged management of combat-related extremity vascular injuries with extensive soft-tissue defects. This approach is associated with the preservation of vascular reconstructions and limbs, low infection and amputation rates, the mitigation of erosion-related bleeding, and timely wound closure. Prospective multicenter studies are needed to optimise and standardise NPWT protocols in this setting.

## Introduction

Extremity vascular injuries account for 12–14% of all combat-related injuries, a figure significantly higher than that reported in previous wars and armed conflicts [1]. This increase is due primarily to two factors. First, high-energy weapons, such as FPV drones, mortars, and improvised explosive devices (IEDs), are widely used. Second, advances in personal protective equipment, including body armour and Kevlar elements, have improved protection for the torso and groin. While these protective measures have reduced the incidence of thoracoabdominal injuries, the extremities remain highly vulnerable to penetrating trauma [2–4].

**Fig. 1:**
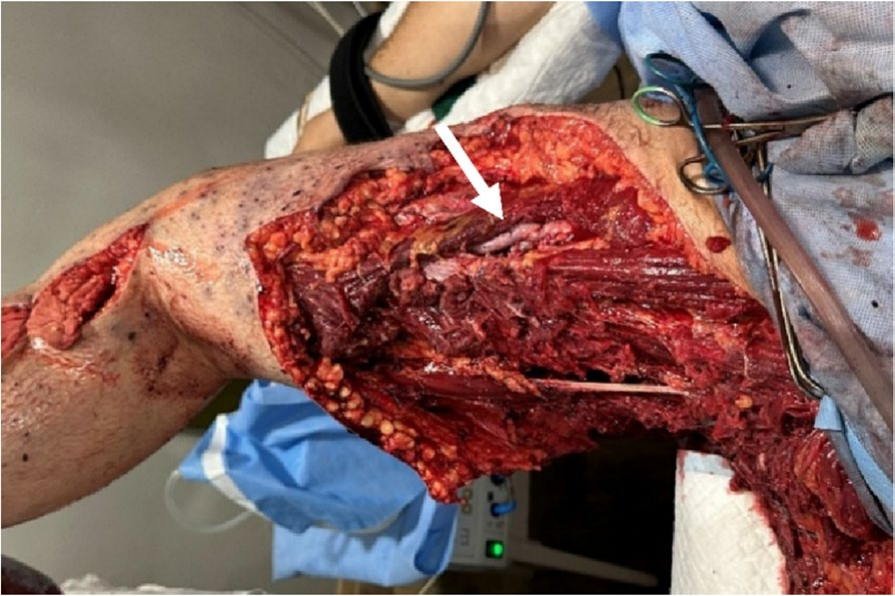
Mine-blast injury to the thigh with massive soft-tissue defect. The absence of tissue coverage over the vascular graft (indicated by the arrow) poses a high risk of erosion-related bleeding

Blast injuries are frequently associated with severe tissue destruction, extensive soft-tissue defects, and high levels of microbial contamination (Fig. 1). These factors greatly complicate postoperative care, increase the risk of infectious complications, and often lead to secondary amputations [5, 6]. The management of such complex injuries requires a multidisciplinary approach and staged surgical interventions, including radical debridement of devitalised tissue and removal of foreign bodies, followed by delayed wound closure [7, 8]. Primary wound closure is generally contraindicated due to zones of secondary tissue injury and a high risk of wound infection [8].

Over the past two decades, negative pressure wound therapy (NPWT) has been increasingly integrated into military trauma surgery. It is now regarded as a standard of care for complex traumatic wounds [9, 10]. NPWT has been shown to reduce the bacterial load, improve wound bed preparation, and promote granulation tissue formation, thereby creating optimal conditions for definitive wound closure [10, 11]. It can be effectively utilized at forward surgical facilities and even during medical evacuation, facilitating wound stabilization and improving patient outcomes [12].

However, the use of NPWT for extremity vascular injuries remains controversial. Concerns persist regarding the potential effects of negative pressure on the vascular wall and anastomotic sites [5, 10, 13]. When extensive soft-tissue loss and microbial contamination are present at the site of vascular reconstruction, there is an increased risk of vessel wall desiccation and erosion-related bleeding. Current clinical guidelines lack specific recommendations for such cases, leaving surgeons without clear guidance and making the selection of an optimal treatment strategy particularly challenging [10, 13]. This evidence gap became particularly apparent during the full-scale invasion of Ukraine, when vascular injuries with soft-tissue loss became increasingly frequent.

This study aimed to evaluate the safety and efficacy of a standardised two-layer NPWT protocol in the management of combat-related extremity vascular injuries with extensive soft-tissue defects during the war in Ukraine.

## Methods

### Patients and data sources

In this retrospective study, we analysed the treatment of 85 patients with severe extremity vascular injuries that were sustained during the war in Ukraine between February and December 2022. NPWT was used in 69 patients (81.2%), all of whom had vascular injuries accompanied by extensive soft-tissue defects overlying the vessels. This subgroup was included in the analysis. There was no control group.

The data presented in this study are not part of a national military trauma registry, as no such registry currently exists in Ukraine. Despite the ongoing conflict for more than a decade, no systematic nationwide studies on combat injuries have been conducted.

This study reflects the experience of a single Role IV surgical facility, the Vascular Surgery Department of the National Military Medical Clinical Center (Kyiv, Ukraine). Detailed prehospital documentation was often unavailable due to wartime conditions. Primary medical records were missing in more than 50% of the cases, and data on tourniquet duration or ischaemia time were incomplete in many instances. Ukraine currently lacks a national trauma registry, which limits the ability to analyse prehospital factors in detail. This retrospective analysis was conducted and reported after institutional approval was obtained on May 26, 2025.

All patients were male active-duty military personnel, with a mean age of 33.4 ± 9.3 years. Comorbidities were rare: one patient had tuberculosis, one had chronic pyelonephritis, and two had active COVID-19 infection at the time of hospitalization.

Clinical data were collected directly by the authors, who were actively involved in surgical care at the Role IV facility, through review of medical records, follow-up of outcomes at Role II–IV facilities, communication with treating surgeons, and analysis of photographic/video materials.

Patients were eligible for inclusion if they met the following criteria: combat injuries involving major arteries or veins of the upper or lower extremities, surgical intervention on a major limb vessel performed at Role II–IV healthcare facilities, the presence of a soft-tissue defect at the site of vascular reconstruction, and the use of NPWT at a Role IV facility as part of postoperative wound management.

Patients were excluded if they had: primary limb amputation performed at a previous level of care, bleeding unrelated to vascular intervention, or wounds older than four weeks complicated by pseudoaneurysm or arteriovenous fistula formation.

### NPWT technique

**Fig. 2:**
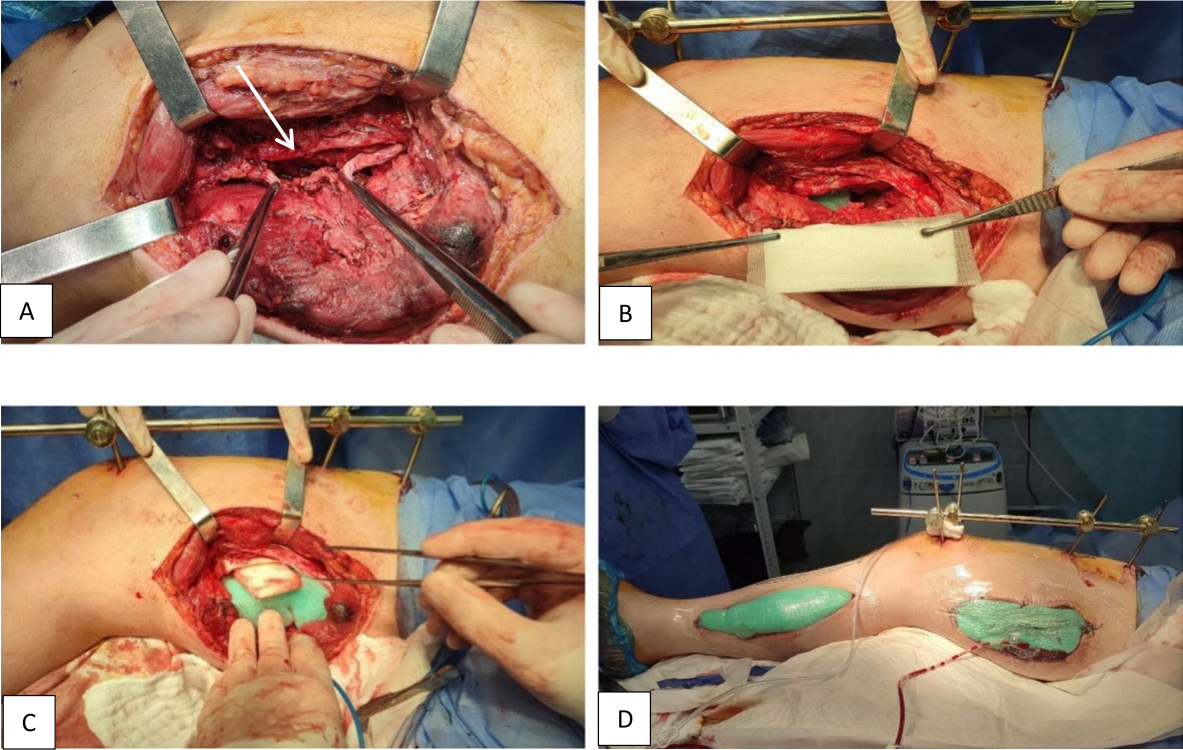
Two-layer NPWT dressing for combat-related femoral vascular injury. **A** The arrow shows the reconstructed superficial femoral artery and the ligated ends of the femoral vein (held with forceps). **B** PVA sponge combined with a silicone mesh serves as a protective barrier placed directly over the vascular repair site. **C** Standard polyurethane foam sponge functions as the second layer for effective drainage. **D** Fully assembled NPWT dressing is ready for application

NPWT was applied after debridement and vascular reconstruction using a two-layer dressing system (Fig. 2). The inner layer consists of a polyvinyl alcohol (PVA) sponge with a nonadherent silicone mesh barrier placed directly over the vascular repair site to protect the vessel wall and anastomoses. The outer layer was standard polyurethane foam. Continuous negative pressure of –70 to –80 mmHg was delivered via portable devices (Genadyne XLR8, Genadyne Biotechnologies, USA; V.A.C. Therapy, 3M, USA). Dressings were changed every 3–4 days or earlier if clinically indicated. This pressure range was chosen based on clinical experience and published reports of increased haemorrhagic/erosive complications with –125 mmHg applied near vascular reconstructions. In this cohort, a pressure of –70 to –80 mmHg provided an optimal balance between exudate evacuation, wound stabilization, and preservation of vascular integrity, while minimising bleeding from fragile, traumatized tissues.

### Outcome measures

The following outcomes were evaluated to assess the efficacy and safety of NPWT: infectious complications, erosion-related bleeding, arterial thrombosis, secondary limb amputation, method of definitive wound closure, number of dressing changes and surgical procedures, and length of hospital stay.

### Outcome definitions

*Infection* was defined as a clinically evident wound infection confirmed by positive wound cultures when feasible and supported by elevated inflammatory markers (C-reactive protein, procalcitonin) or positive blood cultures.

*Thrombosis* was diagnosed clinically (absent pulse, cold extremity, loss of Doppler signal) and confirmed by contrast-enhanced CT angiography when available.

*Erosion-related bleeding* was defined as postoperative haemorrhage originating from the vascular wall or anastomosis in association with purulent-necrotic changes in surrounding tissues, requiring urgent surgical intervention.

We defined *primary closure* as the immediate approximation of native soft tissues (skin and subcutaneous layers, including muscle where applicable) without the use of plastic techniques (flaps or skin grafts).

### Statistical analysis

Statistical analysis was performed using MedStat 5.2 software. Continuous variables are presented as the mean (M), standard deviation (SD), and median (Me). Categorical variables are summarized as absolute numbers (n) and percentages (%). Categorical variables are summarised as absolute numbers (n) and percentages (%), with 95% confidence intervals (CIs) calculated using the Wilson method. Tests of normality and comparative analyses were not performed because this was a purely descriptive study.

## Results

NPWT was integrated into staged surgical management in 69/85 (81.2%) patients with combat-related extremity vascular injuries. The most common mechanism was mine–blast trauma (71%), followed by gunshots (23%) and other explosive mechanisms (6%).

### Vascular injury characteristics

The most frequently affected arteries were the brachial artery (33.3%, *n* = 23), superficial femoral artery (23.2%, *n* = 16), and popliteal artery (11.5%, *n* = 8). Combined arterial and venous injuries were identified in 40% of the patients. Limb fractures were present in 42% of the patients (*n* = 29), contributing to treatment complexity and prolonging the time to definitive wound closure.

### Initial surgical management at forward facilities

As shown in Table 1, arterial flow restoration was achieved in more than two-thirds of the patients through reconstructive procedures. Ligation (10%) and temporary vascular shunting (11%) were performed less frequently, primarily as part of a damage control surgery (DCS) strategy.

**Table 1:** Distribution of surgical interventions for combat-related extremity vascular injuries (97 procedures in 69 patients)

Surgical intervention	Role II	Role III	Role IV	Total
Autovenous grafting	23	12	8	43
End-to-end repair	5	7	2	14
PTFE (polytetrafluoroethylene) grafting	1	1	2	4
Patch angioplasty	3	3	2	8
Ligation	6	1	3	10
Temporary shunt	6	3	2	11
Thrombectomy	2	1	4	7
Total	46	28	23	97

The values are n unless otherwise specified

### Use of PTFE grafts

In four patients, PTFE grafts were used instead of autologous vein conduits. In two cases, this was part of a Role II-III damage-control strategy when vein harvest was not feasible. In two other cases, PTFE grafts were employed during reoperation for erosive bleeding on Role IV. Although autologous material remains the preferred option in contaminated fields, in our experience, PTFE provides satisfactory short-term patency when biological grafts are unavailable.

### Microbiology

Primary wound contamination was documented in 56.5% of patients. The most common pathogens were *Klebsiella* spp. (30.4%) and *Acinetobacter* spp. (17.4%). Microbiological sampling was not performed on 29% of the samples because of a mass-casualty influx.

### Complications

Complications occurred in 19/69 (27.5%) patients (Table 2). Erosion-related bleeding occurred in 9/69 patients (13%; 47.4% of complications); seven episodes were associated with sepsis (*n* = 3) or local infection (*n* = 4). Erosion-related bleeding clustered in two risk windows: days 7–10 (early, typically associated with local infection/partial dehiscence) and days 18–30 (late, characterized by progressive wall erosion/exposure). The median onset date was day 18 (range 6–29). All patients required surgical revision, including autogenous vein graft replacement (*n* = 4), allograft (*n* = 2), ligation (*n* = 2), and arterial wall repair (*n* = 1). No mortality occurred. Arterial thrombosis developed in 6/69 patients (8.7%), revascularization succeeded in five patients, and one patient required amputation. Purulent wound complications developed in 4/69 patients (5.8%), one of whom progressed to below-knee amputation due to progressive phlegmon.

**Table 2:** Complications, interventions, and outcomes among 69 patients managed with NPWT after vascular repair

Type of complication	No. of cases (n)	Key characteristics and interventions	Treatment outcomes
Erosion-related bleeding	9 (5 at Role III, 4 at Role IV)	Median onset: 18 days (range 6–29); recurrent in 3 patients (three episodes each); associated with local infection/sepsis. Surgical management: autogenous vein graft replacement (n = 4), allograft replacement (*n* = 2), vessel ligation (*n *= 2), arterial wall repair (*n* = 1)	Haemorrhage controlled in all cases; no mortality; all limbs preserved
Arterial thrombosis	6 (5 at Role II – III, 1 at Role IV)	Managed with thrombectomy and reconstructive procedures: autogenous vein graft replacement (*n* = 4), primary end-to-end arterial repair (*n *= 2)	Blood flow was restored in 5 patients; 1 patient underwent amputation
Purulent wound complications	4	Managed by repeated debridement and NPWT following wound bed preparation	1 below-knee amputation needed

The values are *n* (%) unless otherwise specified

The rates of erosion-related bleeding, infection, thrombosis, and secondary limb amputation were 13% (95% CI 7.0–23.0), 5.8% (95% CI 2.3–14.0), 8.7% (95% CI 4.1–17.7), and 2,9% (95% CI 0.8–10.0), respectively. These rates are comparable to or lower than those reported in previously published studies of vascular trauma and NPWT use [14–16].

### Wound closure and hospital stay

Definitive wound closure was achieved most often through primary closure (75.4%), followed by split-thickness skin grafting (17.4%), flap techniques (4.3%), and secondary closure (2.9%) (Table 3).

**Table 3:** Definitive wound closure methods among 69 patients managed with NPWT

Method of wound closure	No. of patients (n)	% of total
Primary closure	52	75.4%
Secondary closure (delayed)	2	2.9%
Split-thickness skin grafting	12	17.4%
Flap techniques	3	4.3%

The values are *n* (%) unless otherwise specified

**Fig. 3:**
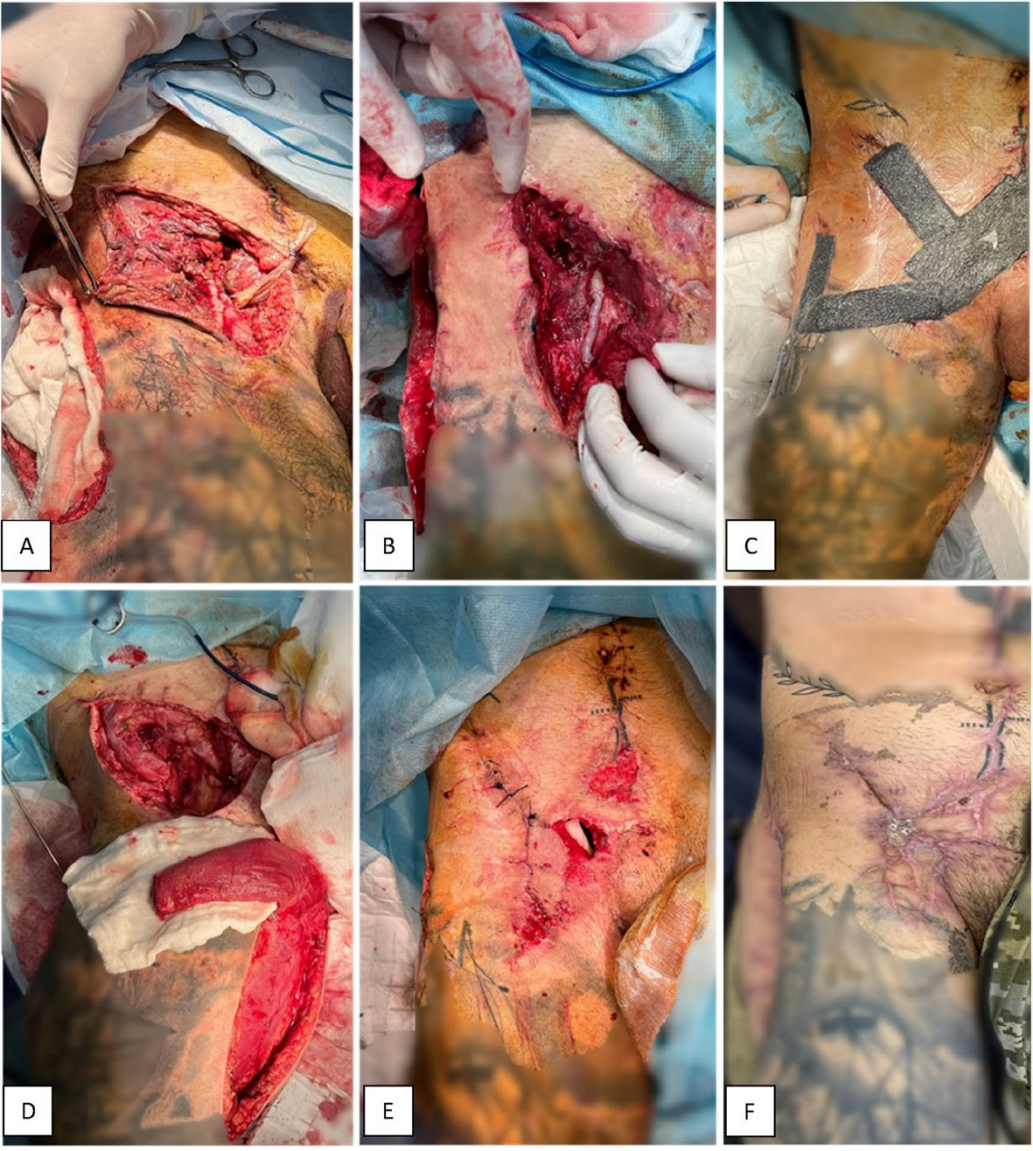
Staged NPWT and flap coverage following femoral artery reconstruction. Identifying marks were obscured to preserve anonymity*.* **A**–**B** Initial wound demonstrates exposed vascular repair with insufficient soft-tissue coverage. **C** NPWT is applied for wound conditioning and infection control. **D** Local muscle flap is prepared to cover the femoral vessels. **E** Inner layer of the two-layer NPWT dressing (PVA sponge) is placed at the wound base to protect the vascular repair. **F** Final wound appearance after complete healing

In patients with soft-tissue defects overlying the site of vascular reconstruction, staged NPWT was used prior to definitive coverage. NPWT was initially applied to reduce microbial contamination and optimise the wound bed for subsequent reconstruction. Once the wound was adequately prepared, coverage was achieved via a local muscle flap, providing reliable protection for vascular repair, preventing vessel desiccation, and supporting limb preservation (Fig. 3). The mean hospital stay was 29.1 ± 11.2 days, with a median of 26 days.

## Discussion

### Main results and context

This retrospective cohort study analysed 69 cases of combat-related limb vascular injuries treated with NPWT at a Role IV facility during the war in Ukraine. All patients presented with extensive soft-tissue defects overlying vascular reconstructions, creating significant challenges for durable coverage and protection of the neurovascular bundle.

These injuries reflect the unique characteristics of modern warfare, which is dominated by high-energy mechanisms such as improvised explosive devices (IEDs), FPV drones, and heavy artillery. In this series, mine-blast trauma accounted for 71% of the cases, which were frequently compounded by prolonged evacuation times and limited availability of air medical transport. Such complex, multi-tissue injuries involving vessels, bone, muscle, nerves, and skin require staged surgical management, beginning with DCS and continuing through definitive limb salvage [17–19].

The findings demonstrate that standardised NPWT using a two-layer dressing (a nonadherent inner barrier over the vascular repair and an outer polyurethane foam) can be safely integrated into staged surgical protocols under battlefield conditions. This approach was associated with a low rate of infectious complications (5.8%), a limb salvage rate of 97.1%, and a secondary amputation rate of 2.9%. These outcomes are particularly notable given the extreme contamination and soft-tissue destruction typical of modern combat trauma.

Importantly, this study provides detailed observations of erosion-related bleeding, a serious but under-reported complication of NPWT in patients with vascular trauma (detailed below). To the best of our knowledge, this represents one of the largest contemporary series of NPWT applications in combat-related vascular injuries, offering new insights into adapting advanced wound management technologies to wartime surgical settings.

### Comparison with previous military experience

The use of NPWT in combat trauma has been widely documented during conflicts in Iraq and Afghanistan [7, 10, 20]. Reports from that period demonstrated that NPWT reduced wound infection rates, accelerated granulation tissue formation, and facilitated delayed closure or flap coverage.

Leininger et al. reported a reduction in infection rates from 80% to 0% when NPWT was combined with pulsed irrigation, with a median time to wound closure of 4.2 days [20]. Peck reported similarly low infection (3.7%) and secondary amputation (2.9%) rates in patients with vascular limb injuries managed using NPWT [21]. Helgeson achieved delayed wound closure in 81% of cases, and Machen described faster granulation and reduced wound size in 286 procedures [22, 23].

Geiger et al. analysed 68 patients who underwent 240 procedures and noted a reduction in the time to closure—from 12 to 4 days—with a limb salvage rate of 93.6% [24]. Stannard and Krug further demonstrated decreased rates of secondary necrosis and improved healing trajectories with NPWT [14, 15].

However, several studies have also reported limitations. Warner reported higher infection rates in NPWT-treated wounds than in those managed with antibiotic beads, underscoring the need for careful wound selection and adjunctive antimicrobial strategies [25]. Älgå et al. found no short-term superiority of NPWT over conventional dressings in smaller wounds, suggesting that its greatest benefit lies in managing large, contaminated defects not amenable to immediate closure [26].

In this context, the present results align with the most favourable historical data. The infection rate (5.8%) and secondary amputation rate (2.9%) in this cohort fall at the lower end of the reported ranges (infection: 3–15%; amputation: 3–12%) [5, 6, 14, 22]. This outcome likely reflects consistent barrier protection over vascular repairs, meticulous debridement, and rigorous wound monitoring during the critical second and third weeks after injury.

To further contextualise these outcomes, a comparative summary of key complications across published series is presented in Table 4.

**Table 4:** Comparison of complication rates in combat-related extremity injuries managed with NPWT

Study/Source	Patients (n)	Infection (%)	Thrombosis (%)	Amputation (%)	Bleeding (%)
Leininger, 2006 (Iraq) [21]	77 CEI	0	0	0	0
Peck, 2007 (Iraq/Afghanistan) [23]	134 CEVI	3.7	5.0	3.0	3
Geiger, 2011 (Iraq) [25]	62 CEI	24.2	—	5.8	—
Älgå, 2020 (Afghanistan) [26]	88 CEI	12.0	—	1	40
Shaprynskyi, 2025 (Ukraine) [27]	231 CEI	21.1	—	—	2.63
**Current study** (Ukraine)	**69 CEVI**	**5.8**	**8.7**	**2.9**	**13**

The data are presented as reported in the original publications

Dash (—) indicates data not available

*CEI *Combat extremity injury, *CEVI *Combat extremity vascular injury

Bold values represent complication rates observed in the current study cohort for comparative purposes

However, previously published reports provide limited procedural detail regarding the use of NPWT in vascular trauma. In the series by Peck et al. (2007) [21], NPWT was used as an adjunct in the management of extremity vascular injuries; however, the authors did not specify whether the device was placed directly over vascular repairs or anastomoses, nor did they describe pressure settings or wound characteristics above the reconstructed vessels. The extent of soft-tissue loss and the presence of associated fractures were also not reported. Consequently, while the infection and amputation rates in Peck’s cohort are broadly comparable to the present findings, direct procedural comparisons should be interpreted with caution. The current study extends previous work by providing explicit technical parameters—including pressure settings (− 70 to − 80 mmHg) and protective two-layer configuration—demonstrating the safe use of NPWT directly over vascular reconstructions under battlefield conditions.

When compared with previous military cohorts, the present series demonstrates infection and amputation rates within or below historical ranges, with similar thrombosis rates. Erosion-related bleeding (13%) was identified as a distinct complication rarely reported in earlier studies, likely reflecting the influence of delayed evacuation, multidrug-resistant infection, and limited early coverage capacity during high-intensity combat operations.

### Comparison with civilian vascular surgery

In civilian vascular practice, NPWT is well established for managing graft infections and groin wounds. Numerous studies have shown that NPWT promotes wound healing, supports graft preservation, and reduces infection rates when combined with radical debridement and proper anastomotic protection [28–30].

Dosluoglu et al. reported successful graft salvage without the need for muscle flap coverage via NPWT in infected groin wounds, with a median healing time of 10–12 days [31]. Krug et al. demonstrated that NPWT improves microcirculation and angiogenesis, creating a favourable physiological environment for wound healing and the integration of reconstructive procedures [15].

Moderate continuous suction (− 70 to − 80 mmHg) has been shown to be effective for controlling exudate while minimising vascular complications. In contrast, higher settings (− 125 mmHg), commonly used in civilian wounds, may exert excessive shear stress on fragile anastomoses, thereby predisposing them to bleeding and thrombosis [14, 15].

Our findings support the use of lower-pressure NPWT (− 70 to − 80 mmHg) near vascular repairs, which is consistent with ESVS 2020 guidance and endorses its use as an adjunct to debridement and targeted antimicrobial therapy [16]. In combat environments, however, NPWT frequently serves as a bridge therapy until definitive soft-tissue coverage can be achieved.

### Safety precautions and recommendations

The most serious complication of NPWT in proximity to vascular structures is erosion-related bleeding. In our study, erosion-related bleeding occurred in 13% of patients, typically two to three weeks after injury. Three patients experienced recurrent haemorrhage, thus underscoring the need for close monitoring during this period. All patients were managed surgically without fatalities, demonstrating that prompt recognition and intervention are crucial for lifesaving outcomes.

Arterial thrombosis was the second most common complication (8.7%), with five patients successfully managed by thrombectomy and revision and one patient requiring amputation due to irreversible ischaemia.

The following checklist summarises key precautions and procedural standards for the safe application of NPWT near vascular repairs in combat-related trauma (Table 5):

**Table 5:** Safety checklist for NPWT near vascular repairs

1. Barrier protection: Cover vascular repairs with a PVA sponge and/or a silicone nonadherent membrane.
2. Two-layer configuration: Use an inner protective barrier and an outer polyurethane foam.
3. Pressure/mode: Apply continuous suction at −70 to −80 mmHg; avoid −125 mmHg near vascular repairs.
4. Dressing interval: Changes every 3–4 days, or sooner if strike-through, odour/fever, increasing pain, or device alarms occur.
5. Monitoring windows: Maintain heightened surveillance on days 7–10 and 18–30 (fresh blood in the tubing/canister, sudden output changes, a decrease in haemoglobin, and loss of seal).
6. Immediate action: At any sign of bleeding, stop NPWT, apply direct pressure or clamps, activate the massive transfusion protocol, and proceed to surgical exploration.
7. Anticoagulation: Reassess anticoagulation at each dressing change and correct any coagulopathy.
8. Infection control: Obtain wound cultures when feasible; initiate/adjust broad-spectrum antibiotics with multidrug-resistant (MDR) coverage; perform aggressive debridement as needed.
9. Early coverage: Involve plastic/reconstructive surgeons early; proceed to muscle-flap or definitive coverage once the bioburden is controlled.
10. Documentation/labelling: Record pressure settings, dressing layers, and change times; label dressings “Vascular repair under NPWT.”

These principles align with the recommendations of the European Wound Management Association [32] and the European Society for Vascular Surgery (ESVS, 2020), which advise caution when using NPWT in wounds with pseudoaneurysms, unprotected anastomoses, or unstable haemostasis [15, 16]. We further recommend integrating NPWT into standardised DCS protocols at Role II–IV facilities, along with staff training to ensure proper technique and patient safety.

### PTFE grafts and material selection

Although autologous vein grafts remain the gold standard for vascular repair in contaminated or complex soft-tissue injuries, their availability in combat trauma is often limited by the extent of tissue destruction, previous vein harvest, or unstable patient conditions. In our series, PTFE grafts were used in four patients—two as part of a Role II damage-control approach and two during reoperations for erosive bleeding. Despite the theoretically increased risk of infection associated with prosthetic materials, short-term outcomes are satisfactory, with no early graft loss. These observations align with previous reports from both civilian and military settings, suggesting that PTFE may serve as a viable temporary option when biological conduits are unavailable.

### Erosion-related bleeding

Erosion-related bleeding remains a major complication in the management of combat-related vascular injuries. In this cohort, the incidence reached 13%, reflecting the combined effects of anatomical, microbiological, and operational factors unique to modern warfare.

Extensive soft-tissue destruction caused by high-energy weapons—such as cluster munitions, mortars, and improvised explosive devices—was frequently accompanied by severe microbial contamination, complex fractures, and concomitant vascular–neural injury. Prolonged or inappropriate tourniquet use, particularly during the early phases of conflict, exacerbates ischaemia and tissue necrosis. Limited access to advanced haemostatic materials and blood products under austere battlefield conditions further complicated haemorrhage control [33, 34].

Evacuation delays due to tactical constraints, including the impossibility of air evacuation under enemy fire and the dominance of opposing air-defence systems, prolonged ischaemia and increased risk of local complications. The lack of timely plastic or flap coverage for soft-tissue defects over vascular repairs further predisposes patients to erosion and thrombosis. This issue was often linked to limited availability of reconstructive surgeons or insufficient reconstructive training among general and vascular surgeons at Role II–IV facilities [35, 36].

Persistent infections with multidrug-resistant organisms—most commonly *A. baumannii, K. pneumoniae, and P. aeruginosa—*play important roles in vascular wall degradation and delayed wound healing. These pathogens produce biofilms and proteolytic enzymes that destroy endothelial and perivascular tissues, leading to anastomotic erosion, graft failure, and recurrent haemorrhage [37–39]. Observations from recent conflicts have linked these infections to delayed wound closure and repeated bleeding episodes [40–42].

Collectively, these factors create a clinical environment highly conducive to the development of erosion-related vascular complications. Prevention of erosion-related bleeding requires aggressive debridement of infected and necrotic tissue, early coverage of the vascular bundle with viable soft tissue, and application of continuous NPWT at − 70 to − 80 mmHg in cases of extensive soft-tissue loss. To minimise pressure-induced vascular injury, a two-layer dressing configuration with a nonadherent inner barrier and an outer drainage foam should be used. This technique allowed preservation of vascular repairs and successful limb salvage in all affected patients.

**Fig. 4:**
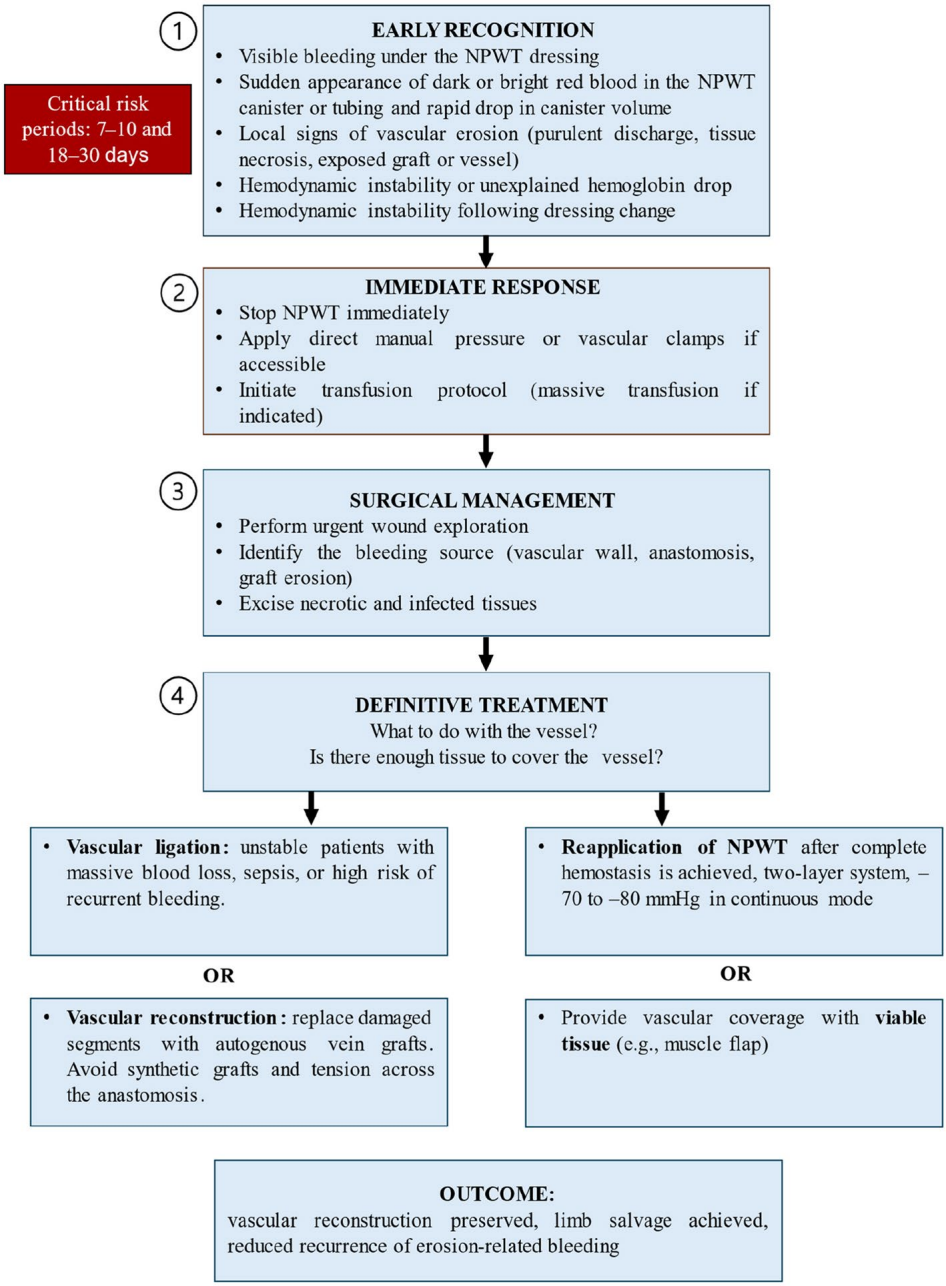
Algorithm for management of erosion-related bleeding in combat-related vascular trauma. Critical risk periods highlighted (days 7–10 and 18**–**30)

To improve outcomes in this high-risk subgroup, we developed a stepwise management algorithm for erosion-related bleeding (Fig. 4). Early detection during two critical risk periods (days 7–10 and 18–30), prompt haemostatic control, targeted surgical revision, and staged NPWT reapplication under reduced suction are emphasized. The adoption of this algorithm within combat trauma systems may enhance repair preservation and reduce secondary amputation rates.

### Microbiological issues in modern combat trauma

Combat wounds differ fundamentally from civilian injuries due to primary contamination, delayed evacuation, and subsequent nosocomial colonisation. In this study, microbial contamination was documented in 57% of cases.

The microbiological profile evolved dynamically:

- First week: predominance of gram-positive organisms (~ 74%).
- Third week: transition toward multidrug-resistant gram-negative bacteria, particularly *Acinetobacter baumannii* (53%) and *Pseudomonas aeruginosa* (15%).

This pattern mirrors findings from previous conflicts [43, 44]. Gram-negative pathogens exhibit high virulence and produce proteolytic enzymes capable of degrading vascular and perivascular tissues. Persistent infection with *A. baumannii*, *K pneumoniae*, or *P. aeruginosa* has been associated with vascular wall destruction, graft.

Erosion, and delayed healing [16, 45]. These microorganisms form biofilms that resist host immunity and antibiotics, contributing to recurrent bleeding and infection persistence [37–39].

NPWT may mitigate the bacterial load by continuously evacuating exudate and maintaining a controlled environment, although it cannot replace targeted antimicrobial therapy. Awareness of shifting microbiological patterns is therefore essential for appropriate empiric antibiotic selection and infection control in combat-related vascular trauma [7, 8, 15, 43].

### Operational implications for military care (Roles II–IV)

These findings have important implications for modern military medicine. In resource-limited battlefield environments, structured wound management and early interdisciplinary coordination are crucial for optimising outcomes and maintaining continuity of care.

1. Indications for NPWT:
  NPWT is particularly valuable when immediate flap or graft coverage is not feasible and when wounds are heavily contaminated. Its capacity to stabilize the wound environment, reduce bacterial burden, and preserve vascular repairs makes it a cornerstone of staged reconstructive strategies.
2. Standardisation of practice:
  Standardisation of NPWT protocols—including dressing configurations, pressure parameters, and change intervals—is essential to ensure procedural safety, reproducibility, and interoperability across evacuation levels.
3. Reconstructive coordination:
  Early coordination with reconstructive or plastic surgeons should be initiated once infection control has been achieved. When such expertise is unavailable, general and vascular surgeons should be trained in fundamental reconstructive techniques to enable timely coverage.
4. Monitoring and complication prevention:
  Close monitoring beyond day 14 is vital, as erosion-related bleeding most commonly occurs during this period. Early recognition of warning signs—such as a sudden change in drainage, loss of seal, or a drop in haemoglobin—can prevent catastrophic bleeding.
5. Infection control principles:
  Infection control in resource-limited settings should prioritise serial surgical debridement, early cultures when feasible, and prompt de-escalation/escalation, with adherence to antibiotic stewardship principles aligned with MDR risk.
6. Cost-effectiveness and system integration:
  Although NPWT systems entail initial costs, they are cost-effective overall because of reduced hospital days, fewer infections, and lower amputation rates.
  The integration of NPWT into standardised DCS algorithms, supported by structured staff training, may further improve outcomes in patients with extremity vascular trauma under combat conditions.

### Limitations and Future Perspectives

This study has several limitations. It was a retrospective, single-centre analysis conducted under active combat conditions without a control group, which restricts generalisability and statistical inference. Prehospital data—such as evacuation times, tourniquet durations, and haemostatic interventions—were incomplete, reflecting operational realities.

Microbiological testing was inconsistent due to resource constraints and mass-casualty conditions, likely resulting in underestimates of contamination rates. Long-term follow-up data were unavailable because most patients were evacuated to other facilities or abroad. These factors highlight the need for structured trauma registries and centralised data collection in military medicine.

Formal injury severity scoring (ISS/NISS) could not be applied in this cohort due to the operational constraints of combat casualty care and the absence of standardised anatomical documentation in many field medical records. Based on clinical characteristics such as the extent of soft-tissue destruction, vascular injury, ischaemia duration, and shock grade, most cases corresponded to severe or critical trauma severity. Future studies should incorporate standardised trauma scoring to improve consistency in outcome analysis.

Despite these constraints, this study provides one of the first structured evaluations of NPWT use in combat-related vascular trauma during high-intensity warfare. The results offer valuable insights into the feasibility, safety, and clinical applicability of NPWT in resource-limited environments. Future research should prioritise prospective, multicentre studies to determine optimal NPWT parameters—pressure settings, dressing materials, and change intervals—and evaluate long-term outcomes such as graft patency, limb function, and quality of life. Integrating NPWT into standardised DCS protocols, supported by continuous training and quality monitoring, may further improve vascular repair preservation and limb salvage.

## Conclusion

NPWT using a two-layer configuration is a safe and effective adjunct in the management of complex combat-related extremity vascular injuries. By maintaining a controlled wound environment, protecting vascular structures from desiccation and mechanical trauma, and reducing infection risk, NPWT supports graft integrity and limb viability in austere wartime conditions.

When integrated into staged surgical protocols with appropriate safeguards, NPWT can reduce complication rates, improve haemostasis, and enhance outcomes in damage-control surgery for vascular trauma. Standardised NPWT implementation across military medical echelons could strengthen limb-salvage efforts and improve the continuum of care from the point of injury to definitive reconstruction.

## Data Availability

The datasets generated and/or analysed during the current study are not publicly available owing to military confidentiality restrictions but are available from the corresponding author upon reasonable request.

## Abbreviations

CTA: Contrast-enhanced CT angiography
DCS: Damage control surgery
ESVS: European Society for Vascular Surgery
IED: Improvised explosive device
ESBL: Extended-spectrum β-lactamase
NPWT: Negative pressure wound therapy
PTFE: Polytetrafluoroethylene
PVA: Polyvinyl alcohol
